# Expectations of Mental Illness Disclosure Outcomes in the Work Context: A Cross-Sectional Study Among Dutch Workers

**DOI:** 10.1007/s10926-022-10026-x

**Published:** 2022-02-08

**Authors:** I. E. van Beukering, M. Bakker, P. W. Corrigan, S. Gürbüz, R. I. Bogaers, K. M. E. Janssens, M. C. W. Joosen, E. P. M. Brouwers

**Affiliations:** 1 Tranzo, Scientific Center for Care and Wellbeing, Tilburg School of Social and Behavioral Sciences, Tilburg University, P.O. Box 90513, 5000 LE Tilburg, The Netherlands; 2 Netherlands Labour Authority, Den Haag, The Netherlands; 3 Department of Methodology and Statistics, Tilburg School of Social and Behavioral Sciences, Tilburg University, Tilburg, The Netherlands; 4 Department of Psychology, Illinois Institute of Technology, 3424 South State Street, Chicago, IL 60616 USA; 5 Brain Research and Innovation Centre, Ministry of Defense, Amsterdam, The Netherlands

**Keywords:** Mental illness, Disclosure, Expectations, Work, Discrimination

## Abstract

*Purpose* The decision whether to disclose mental illness at work can have important positive and negative consequences for sustainable employment and well-being*.* The aim of the study is (1) to examine workers’ expectations of outcomes of mental illness disclosure in the workplace and to evaluate their expectations regarding which factors are of influence on these outcomes, (2) to identify distinct subgroups of workers, and (3) to characterize these subgroups in terms of personal, sociodemographic, and work-related characteristics. *Methods* In this cross-sectional survey study, a sample of 1224 Dutch workers was used. Latent Class Analysis (LCA) was used to identify classes of workers based on expected workplace mental illness outcomes. A three-step approach LCA was chosen to investigate whether the classes differed in characteristics. *Results* The majority of workers expected predominantly positive outcomes of workplace mental illness disclosure (e.g., being able to be one’s authentic self; 82.4%), even though they simultaneously expected disclosure to lead to advancement-related discrimination (e.g., lower chances of contract renewal; or getting a promotion; 68.4% and 57%, respectively). Six distinct subgroups of workers were identified based on expected workplace mental illness disclosure outcomes: two positive classes (50.1%), two negative classes (33.3%), and two classes who indicated not to know what the outcomes would be (16.7%). Significant differences between the classes were found on personal experience, work-related association with mental illness, gender, educational level, and workplace atmosphere. *Conclusion* The disclosure process is complex, as most workers were optimistic (i.e., expected generally positive outcomes) whilst simultaneously expecting workplace discrimination. Subgroup differences in expectations regarding workplace mental illness disclosure outcomes were found.

## Introduction

The decision whether or not to disclose mental illness at work can have important positive and negative consequences for sustainable employment and well-being. Disclosure can be a facilitator and a barrier for finding and maintaining work. It can lead to work adjustments and social support [1–3]. In general, disclosure can increase well-being [1, 4]. Also other studies that were not particularly related to the workplace showed that disclosure can lead to acceptance and support [5], and, after completing the Coming Out Proud program, decreased self-stigma and diminished depression [6]. Nevertheless, disclosure can also be a barrier as it can lead to adverse employment outcomes due to stigma and discrimination, such as problems obtaining a job [1, 7–10], job loss [8, 11], limited advancement opportunities [8, 12], and harassment at work [13, 14]. Also outside the workplace mental illness disclosure can lead to increased self-stigma, shame, harm and discrimination [5]. Just like disclosure, non-disclosure can also be both a facilitator and a barrier for finding and maintaining work. It can avoid stigma and discrimination, and enable workers to maintain a positive status quo at work [15]. For example, people who were more reluctant to disclose mental illness during a job interview were more likely to be reemployment than people who were less reluctant to disclose mental illness [16]. However, non-disclosure can also be a barrier to sustainable employment because of job loss due to not receiving necessary work accommodations [17, 18].

While most people may have a preference for disclosure, it may make them vulnerable to stigma and discrimination. For example, two recent studies on workplace mental illness disclosure showed that 73% of Dutch workers with mental illness disclosed to their managers [19], and 75% of Dutch workers without mental illness indicated they would disclose to their managers [20]. Another recent study showed that 64% of Dutch line managers were reluctant to hire people with mental illness [7]. This contrast illustrates the tension between the preference for disclosure and possible negative employment outcomes.

It is important that people with mental illness participate in the labour market, as they could also benefit from the positive effects of employment, such as social contact, routine, and structuring time [21, 22]. Unemployment is known to have a negative impact on health, such as depression and psychological distress [23]. In addition, unemployment has been associated with societal issues, like poverty and increased cost for society [24, 25].

Although the decision whether to disclose or not to disclose a mental illness is a very complicated and important one, research about disclosure decision processes, especially on the expectations of mental illness disclosure outcomes in the work context, is scarce. Multiple studies have shown that actual disclosure decisions were based on anticipated negative outcomes [2, 15, 26], but fewer studies have been conducted on the role of anticipated positive outcomes on disclosure decisions [15]. Furthermore, research has shown that expected workplace disclosure outcomes differ between multiple stakeholder groups (people with mental illness, human resources professionals, employers, work reintegration professionals and mental illness advocates) [1]. Expected disclosure outcomes may therefore also be perceived differently between subgroups of workers. In addition, little is known about how certain factors influence the disclosure process, such as the roles of workplace climate, educational level, gender and age. In sum, more research is needed to look for mechanisms and factors related to the disclosure decision process, which ultimately can help to make people more resilient to stigma and protect them against adverse occupational outcomes such as job loss [27]. For instance, two small scale studies have shown that providing support to people with mental illness in managing their workplace disclosure communication led to significantly higher employment rates after 6–12 weeks [28, 29]. However, larger-scale and longitudinal intervention studies on the effect of the disclosure decision for sustainable employment are scarce, as is more fundamental research [30]. As yet, little is known about the decision making process, and the current study aims to add to a better understanding of this process by investigating workers’ expectations.

Specifically, the aim of this explorative study is to examine: (1) workers’ expectations of workplace mental illness disclosure outcomes, and to evaluate their expectations regarding which factors are of influence on disclosure outcomes (2) to identify distinct subgroups of workers based on their expectations of workplace mental illness outcomes, and (3) to characterize these subgroups in terms of personal, sociodemographic, and work-related characteristics.

## Methods

### Study Design and Participants

For this cross-sectional survey study, data were collected in February 2018 in the Longitudinal Internet Studies for the Social Sciences (LISS) panel [31]. CentERdata, a Dutch research institute specialized in data collection, administers the LISS panel. The panel consists of a representative and random selection of Dutch addresses of 5,000 households, including 7,357 panel members who participate in monthly internet surveys covering different domains like work, education, housing, income, time use, political views, values, and personalities. Prior to participation in the panel, members gave informed consent to participate in the surveys. For more information about the LISS-panel, see www.lissdata.nl.

An online questionnaire, was sent to 1,671 Dutch adults who were part of the panel, had paid jobs and were not working in management positions. The Ethics Review Board of the School of Social and Behavioral Sciences of Tilburg University gave Ethical approval for this study (registration number: RP193). The Strengthening the Reporting of Observational Studies in Epidemiology guidelines were followed during the reporting of this study [32].

### Research Context

In the Netherlands workers with disabilities are protected by the Gatekeeper Improvement Act and the Extended Payment of Income Act. The responsibility for reintegration and benefits lies with the employer as well as with the worker and the occupational physician when sickness absence occurs [33]. Employers compensate the sickness absence for two years by paying at least 70% of the salary [34]. Moreover, employers are prohibited to ask about the health problems of a worker. When workers drop out due to sickness, workers have to consult an occupational physician (OP). The OP is hired by the employer and conducts an independent assessment which results in collaboration with the worker in a reintegration plan, including reasonable accommodations. In the reintegration phase, the worker meets frequently with the OP for advice to promote to return to work.

### Measures

A new questionnaire was designed to address the aims of this study. First, literature on stigma, discrimination, and mental illness in the workplace was searched. The main themes of the questionnaire were determined based on the existing literature. Second, after the literature was searched and items for the questionnaire were developed accordingly, the items were discussed with senior researchers and international experts on stigma and mental illness, to modify and improve the questionnaire. Third, the questionnaire was pilot tested among workers (N = 18) within the researchers’ network and again adjusted based on feedback, resulting in the final version. In this study the following items from the questionnaire were used:

- Expectations of workplace mental illness disclosure outcomes were measured using a set of 15 items on how likely a respondent considers certain consequences to be if a worker with mental illness issues disclosed to others in the workplace. The items were based on existing literature on workplace disclosure [1, 17, 35], and the response options were 1 (very unlikely), 2 (unlikely), 3 (likely), 4 (very likely) and 5 (I don’t know). The items addressed the following topics: (1) improved relationships, (2) social behavior of colleagues, (3) the ability to be authentic, (4) friendly workplace culture, (5) work environment support, (6) improved work functioning, and (7) advancement-related work discrimination or opportunities.
- Factors of influence on disclosure were measured by asking employees to what extent they would advise a good friend with mental illness whether to tell his or her manager about it. This set of 14 items was adapted from the Brohan, et al. [17] and Dewa [35]. The ordinal response options were 1 (certainly don’t tell), 2 (better not tell), 3 (better do tell), and 4 (certainly do tell).
- The personal, sociodemographic, and work-related characteristics, which were expected to be associated with stigma [1, 15, 18] and/or were also included in other disclosure literature [7, 19, 20] were also retrieved via the questionnaire. Personal variables included personal experience with dealing with coworkers with mental illness in the workplace, personally having (had) a mental illness, disclosure of mental illness to a line manager, familiarity with people with mental illness (other than respondent self), personal favorable experience with workers with mental illness, and association with types of mental disorders when thinking of an employee with mental illness. Sociodemographic variables were age, gender, and educational level. The work-related characteristics used were workplace atmosphere, size of the company, type of industry, and gross income per month.

### Statistical Analysis

To examine Dutch workers’ expectations of workplace mental illness disclosure outcomes and to evaluate their expectations regarding which factors are of influence on disclosure outcomes (Research aim 1), descriptive statistics were used. For the items on outcome expectations, expectations of workplace mental illness disclosure outcomes were converted into 0 (unlikely), 1 (unknown), and 2 (likely). Furthermore, an extra exploratory analysis (i.e., descriptive statistics) was added to provide knowledge on the factors of influence on disclosure. Therefore, the factors of influence on disclosure were converted into 0 (non-disclosure) and 1 (disclosure).

For the identification of subgroups based on outcome expectations of mental illness disclosure (Research aim 2), Latent Class Analysis (LCA) was used. For the LCA, unlike for the descriptive statistics, the scores of expectations of the negative expected workplace mental illness disclosure outcomes were converted with higher scores indicating more positive expected consequences. The LCA consisted of the following steps.

First, a latent class model was built using the items on expectations of workplace mental illness disclosure as categorical indicators, which were converted in the previous step to three categories (unlikely, unknown, likely). To identify the most suitable number of classes, multiple fit indices were used in combination with a subjective evaluation of the models’ relevance. The models were evaluated based on the following goodness-of-fit indices the Bayesian information criterion (BIC), the Akaike information criterion (AIC) and with 3 as penalizing factor (AIC3). To determine the optimal number of classes the BIC is the best performing goodness-of-fit indices [36]. The optimum model is the one with the lowest BIC. Although a lower AIC and AIC3 also indicate a better fit of the model. Moreover, the entropy of the model and the improvement of model fit when adding an extra class tested by bootstrap likelihood ratio tests (BLRT) were also included in determining the optimal numbers of classes. The bivariate residuals were examined to determine if the assumption of local dependencies was violated between the items of the classes. The assumption is violated when the bivariate residuals are higher than 10, to take multiple testing into account. The model is modified by stepwise adding direct effects with the variables that have large bivariate residuals. The smallest class had to meet the minimum requirement of 5% of the total sample size [37].

Second, the employees were assigned to the class with the highest posterior probability. This means that workers were placed in the class that suited them the best.

Third, to characterize these subgroups in terms of personal, sociodemographic, and work-related characteristics (Research aim 3), a three-step approach LCA was chosen to investigate whether the classes differed in: (1) personal; (2) sociodemographic; and (3) work-related characteristics. Therefore, personal experience with dealing with coworkers with mental illness in the workplace was merged into the categories ‘negative’, ‘neutral’, ‘positive’ and ‘none’, and the variables concerning disclosure of a mental illness asked both workers with and without mental illness were merged into ‘no disclosure/no mental illness’ and ‘disclosure’. Familiarity with people with mental illness was converted to ‘not familiar’, ‘little familiar’ and ‘very familiar’. Association with types of mental disorders when thinking of an employee with mental illness was converted three dichotomous variables: association with work related mental disorders, association with common mental disorders, and association with other mental disorders. Educational level was merged into ‘low’ (primary school, intermediate secondary, US: junior high school), ‘secondary’ (higher secondary education/preparatory university education, US: senior high school; intermediate vocational education, US: junior college), and ‘high’ (higher education, US: college; university). Workplace atmosphere was merged into ‘negative’, ‘neutral’ and ‘positive’. Size of the company was merged into ‘small’ (≤ 49 workers) and ‘medium/large’ (> = 50 workers), and type of industry was dichotomized into ‘private’ and ‘public’. All 1,224 respondents were included. Because workplace characteristics size of the company, type of industry and gross income per month had missing values, these missing data were imputed using Latent GOLD’s imputation procedure [38]. The significant differences between the classes regarding the characteristics were determined using Wald tests (p < 0.05), these tests were also used to see which pairs of classes differed.

The statistical analyses were conducted using SPSS version 24 for descriptive analyses, and Latent GOLD 5.1 for LCA.

## Results

The online questionnaire was filled out by 1224 respondents (response rate = 73.5%), 72.6% of them indicated that they had never experienced mental illness. The workers (57.2% female) had a mean age of 45 (SD = 12.1) years, and had mostly a secondary (40.1%) or higher (42.8%) educational level. Other characteristics of the sample can be found in Table 1.

**Table 1:** Characteristics of the sample

	%	M (SD)
Personal characteristics		
Having/had a mental illness (N = 1224)		
No	72.6	
Yes	27.4	
Sociodemographic characteristics		
Age in years (N = 1224)		44.6 (12.1)
Gender (N = 1224)		
Male	42.8	
Female	57.2	
Educational level (N = 1224)		
Low	17.1	
Secondary	40.1	
High	42.8	
Work-related characteristics		
Type of industry (N = 972^a^)		
Private	56.1	
Public	43.9	
Size of the company (N = 742^a^)		
Small (up to 50 workers)	45.8	
Medium/large (more than 50 workers)	54.2	
Gross income per month (in euro) (N = 1117^a^)		4855 (2384)

^a^Information not available for all workers

### Research Aim 1: To Examine Dutch Workers’ Expectations of Workplace Mental Illness Disclosure Outcomes and to Evaluate Their Expectations Regarding Which Factors are of Influence on Disclosure Outcomes

Expected mental illness disclosure outcomes and influences on disclosure as viewed by the sample of N = 1224 workers are depicted in Table 2. As can be seen, respondents expected both positive and negative outcomes of workplace disclosure. Regarding positive expectations, most (82.4%) respondents believed disclosure would improve worker well-being because it would enable the worker to be his/her true, authentic self. In addition, 77.2% expected that disclosure would lead to manager willingness to realize work adjustments (77.2%), and that it would decrease the chances of long-term sickness absence (76.2%). Most prevalent negative expectations were that disclosure during a temporary contract would decrease the likelihood of contract renewal (68.4%), that it would diminish the worker’s chances to be promoted to a higher position in the future (56.6%) and that it would increase the likelihood that the employer would want to get rid of the worker (43.4). Furthermore, it is noteworthy that about one in four respondents believed workplace disclosure would lead to the worker being liked less and to be included in social activities less often (25.2% and 23.4%, respectively).

**Table 2:** Expected mental illness disclosure outcomes and influences on disclosure (N = 1224)

		%		
	Expected mental illness disclosure outcomes	Unlikely	Unknown	Likely
Positive	Disclosure will improve well-being because the worker can be his/her true, authentic self	6.5	11.1	82.4
	Disclosure will lead to manager willingness to make work adjustments if necessary	7.6	15.2	77.2
	Disclosure will decrease the chances of long-term sickness absence	10.8	13.0	76.2
	Colleagues will understand and will be prepared to take over work tasks	12.5	12.7	74.8
	Disclosure will contribute to a friendly work culture where employees feel good	17.2	20.4	62.3
	Disclosure will improve relationships with colleagues	19.7	21.2	59.1
	A worker’s disclosure to a line manager will improve their relationship	21.2	24.0	54.8
	Workplace disclosure will enable the worker to perform better at work	28.6	17.7	53.7
Negative	If the worker has a temporary contract, disclosure will decrease the likelihood of contract renewal	12.2	19.4	68.4
	Disclosure will diminish the worker’s chances of being promoted to a higher position in the future	22.0	21.4	56.6
	It will increase the likelihood that the employer will want to get rid of this worker	30.9	25.7	43.4
	Disclosure will lead to unpleasant reactions of others (gossip, jokes)	36.1	21.9	42.0
	Colleagues will be less inclined to work with the worker	43.9	23.3	32.8
	The worker will be liked less	52.2	22.6	25.2
	Colleagues will be less inclined to invite the worker to social activities outside the workplace	57.0	19.5	23.4

	Respondents’ disclosure advice to a friend with mental health problems, under different circumstances (Influences on disclosure)	Non-disclosure	Disclosure	
	If (temporary) work adjustments could help the worker	6.7	93.3	
	If it is clear to see that the worker is not doing well	9.5	90.5	
	If the worker has a good relationship with his/her manager	10.2	89.8	
	If the worker has a job where any mistakes on his/her part will not have serious consequences	16.8	83.2	
	If the worker generally likes to talk about his/her feelings and/or personal life with others	17.8	82.2	
	If the worker describes the atmosphere at work as friendly and/or positive	21.5	78.5	
	If the manager is female	26.5	73.5	
	If the manager is male	29.5	70.5	
	If the worker has a disorder for which other people often show little understanding	41.2	59.8	
	If the worker cannot afford to run the financial risk of losing job	41.7	59.3	
	If the mental problems will not affect work performance	32.6	57.4	
	If few suitable jobs are available due to an economic recession	52.5	47.5	
	If the worker has a temporary contract that will end soon and might be renewed	59.1	40.9	
	During a job application interview with an employer who the worker doesn’t know yet	65.4	34.6	

When evaluating which factors were expected to be of influence on the disclosure outcome, it was found that respondents’ advice was predominantly positive: Table 2 shows that in eleven out of fourteen given conditions, workers would advise a good friend positively on disclosing mental illness to a line manager. For example, almost all workers would advise disclosure if work adjustments could help the worker (93.3%), if it is clear to see that the worker is not doing well (90.5%), or if the worker has a good relationship with their manager (89.8%). Only in three of the potential situations, respondents’ views were more divided. The majority of respondents advised non-disclosure during a job application interview with an employer who the worker doesn’t know yet (65.4%), if the worker has a temporary contract that will end soon and might be renewed (59.1%), and if few suitable jobs are available due to economic recession (52.5%).

### Research Aim 2: Identifying Subgroups of Workers Based on Their Expectations of Workplace Mental Illness Disclosure Outcomes

Within the LCA, multiple fit indices were used to identify the most suitable number of classes. Fit statistics for latent classes 1–10 are presented in Table 3. BIC had the lowest value in the eight-class solution. The results of the BLRT continued to improve, suggests that adding ninth or tenth class fits the data significantly better. While fit estimates did improve with the addition of a ninth class, reductions in AIC and AIC3 were small. However, the solutions with seven or more clusters did not meet the minimum requirement of 5% of the total sample size for the smallest latent class. Compared to the other solutions which did meet this minimum requirement, the six-class solution had the lowest BIC, AIC, and AIC3. Therefore, the six-class solution was selected. The six-class solution had high entropy (0.87) which indicates a good separation of the classes. Within the six-class solution three pairs of variables had large bivariate residuals, therefore direct effects were added between the variables: *improved relationship with the line manager* and *improved relationships with colleagues, employer wants worker to stay* and *increased promotion chance*, and *renew temporary contract* and *increased promotion chance*. This resulted in a lower BIC, AIC, and AIC3 (BIC = 25,347.91; AIC = 24,341.27; AIC3 = 24,538.27). After examination of the fit indices followed by a subjective evaluation of the models’ relevance, adding extra classes did not lead to a more relevant representation of the classes, the six-class solution provided the best representation of the classes in the data.

**Table 3:** Fit indices for latent class analysis (N = 1224)

Model	LL	BIC	AIC	AIC3	Npar	Df	P value BLRT	Entropy R^2^
1-Cluster	− 16,673,248	33,559,793	33,406,497	33,436,497	30	1194	0.000	–
2-Cluster	− 14,296,688	29,027,078	28,715,375	28,776,375	61	1163	0.000	0.920
3-Cluster	− 13,280,713	27,215,535	26,745,426	26,837,426	92	1132	0.000	0.888
4-Cluster	− 12,767,201	26,408,918	25,780,403	25,903,403	123	1101	0.000	0.900
5-Cluster	− 12,457,432	26,009,785	25,222,863	25,376,863	154	1070	0.000	0.885
6-Cluster	− 12,275,281	25,865,890	24,920,562	25,105,562	185	1039	0.006	0.870
7-Cluster	− 12,132,601	25,800,935	24,697,202	24,913,202	216	1008	0.012	0.870
8-Cluster	− 12,013,512	25,783,163	24,521,023	24,768,023	247	977	0.010	0.876
9-Cluster	− 11,923,138	25,822,823	24,402,277	24,680,277	278	946	0.024	0.877
10-Cluster	− 11,830,282	25,857,516	24,278,563	24,587,563	309	915	0.016	0.877

*LL* log likelihood, *BIC* Bayesian information criterion, *AIC* Aikake information criterion, *AIC3 *Aikake information criterion 3, Npar numbers of para-meters, *BLRT* bootstrap likelihood ratio test

**Fig. 1:**
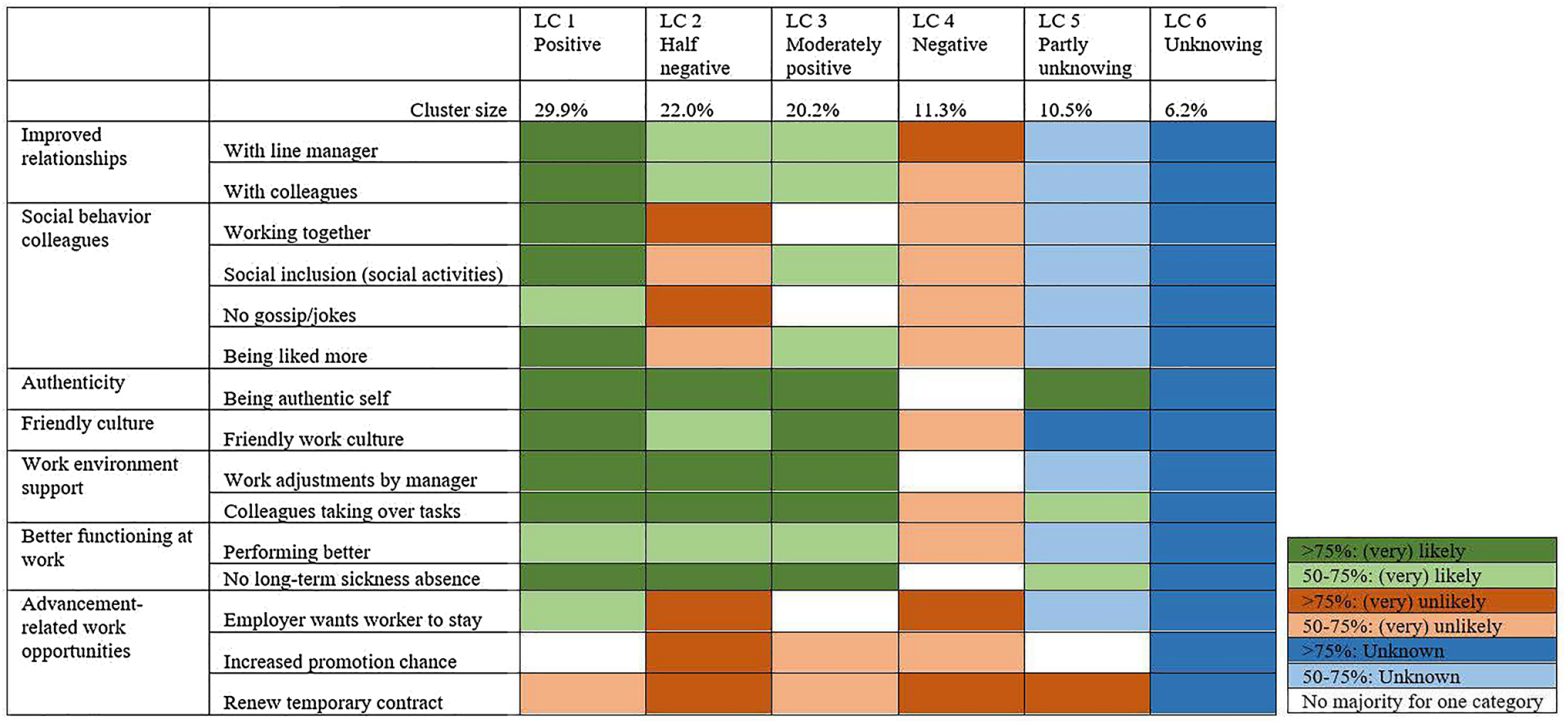
Profiles of the six classes based on expected consequences of disclosure at work

Figure 1 presents the six classes of workers and their expectations of workplace mental illness disclosure outcomes. The figure shows for each class whether a majority (50–75%) or a large majority (> 75%) of workers expected the named workplace mental illness disclosure outcomes were likely (green), unlikely (red), or unknown (blue). White cells indicate that within that class there was no majority of workers who expected the outcome to be either likely, unlikely, or unknown.

The workers in LC 1 and LC 3 expected mostly positive mental illness disclosure outcomes. The *positive* class (LC 1; 29.9% of the workers) expected predominantly positive consequences of disclosure. However, even in this positive class workers expected that there is an increased chance of no renewal of a temporary contract. The *moderately positive* class (LC 3; 20.2% of the workers) also expected mainly positive outcomes of disclosure at work, although with slightly lower probabilities. However, these workers expected that disclosure would lead to an increased chance of no renewal of a temporary contract and diminished promotion chances.

The workers in LC 2 and LC 4 expected (partly) negative mental illness disclosure outcomes. The *half negative* class (LC 2; 22.0% of the workers) expected negative outcomes when it comes to social behavior of colleagues and advancement-related work opportunities. Within this class a majority expected positive consequences like improved relationships, authenticity, friendly culture, work environment support and better functioning at work. The *negative* class (LC 4; 11.3% of the workers) consists of workers who predominantly expected negative mental illness disclosure outcomes.

The workers in LC 5 and LC 6 (partly) did not know what consequences to expect of disclosure at work. The *partly unknowing* class (LC 5; 10.5% of the workers) did not know what to expect of more than half of the disclosure outcomes. However, they did expect that renewal of a temporary contract is unlikely. In contrast however, they also expected positive outcomes on authenticity, the willingness of colleagues to take over work tasks and a reduced risk of long-term sickness absence. The *unknowing* class (LC6; 6.2% of the workers) consisted of workers who did not know what to expect of all of the mentioned disclosure outcomes.

### Research Aim 3: Differences in Personal, Sociodemographic, and Work-Related Characteristics

Table 4 summarizes the associations between the personal, sociodemographic, and work-related characteristics covariates and class membership. Significant differences between the six classes were found on personal experience, work-related association with mental illness, gender, educational level, and workplace atmosphere. The most notable differences between the six classes are mentioned below.

**Table 4:** Characteristics of workers with regard to the full sample and the six classes

		Full sample (N = 1.224)	LC 1 Positive (N = 366)	LC 2 Half negative (N = 269)	LC 3 Moderately positive (N = 248)	LC 4 Negative (N = 137)	LC 5 Partly unknowing (N = 128)	LC 6 Unknowing (N = 76)	p-value
Personal characteristics	Having/had a mental illness (MI)								.480
	No	72.6%	70.7%	75.9%	67.2%	77.9%	71.5%	80.6%	
	Yes	27.4%	29.3%	24.1%	32.8%	22.1%	28.5%	19.5%	
	Disclosure of MI								.260
	No disclosure/no mental illness	79.9%	76.1%	84.3%	75.6%	87.3%	80.0%	83.1%	
	Disclosure	20.1%	23.9%	15.7%	24.4%	12.7%	20.0%	16.9%	
	Familiarity with people with MI								.610
	Not familiar	27.3%	22.5%	30.6%	23.6%	25.9%	33.0%	43.5%	
	Little familiar	18.3%	18.8%	21.1%	16.6%	22.9%	14.5%	9.9%	
	Very familiar	54.4%	58.8%	48.3%	59.8%	51.2%	52.5%	46.7%	
	Personal experience with workers with MI								**.001**
	Negative	7.4%	4.4%	10.1%	6.8%	15.0%	4.8%	3.9%	
	Neutral	29.5%	24.1%	33.2%	29.5%	34.2%	32.2%	29.5%	
	Positive	32.0%	45.3%	28.6%	30.0%	19.1%	27.2%	18.4%	
	None	31.1%	26.2%	28.0%	33.6%	31.8%	35.9%	48.2%	
	Association MI: work related disorders								**.010**
	No	28.4%	19.3%	34.9%	25.7%	38.7%	30.7%	36.2%	
	Yes	71.6%	80.7%	65.1%	74.3%	61.3%	69.3%	63.8%	
	Association MI: common disorders								.630
	No	52.5%	52.1%	50.5%	53.4%	52.9%	50.9%	61.5%	
	Yes	47.5%	47.9%	49.5%	46.6%	47.1%	49.1%	38.5%	
	Association MI: other disorders								.910
	No	73.1%	74.0%	71.6%	75.3%	72.6%	70.1%	73.1%	
	Yes	26.9%	26.0%	28.4%	24.7%	27.4%	29.9%	26.9%	
Socio demographic characteristics	Age (years)								.320
	Mean	44.6	45.6	43.8	44.2	45.0	45.3	42.6	
	Gender								**.000**
	Male	42.8%	41.4%	52.2%	32.4%	56.6%	30.5%	46.3%	
	Female	57.2%	58.6%	47.8%	67.6%	43.4%	69.5%	53.7%	
	Educational level*								**.002**
	Low	17.1%	15.2%	20.6%	8.7%	20.3%	22.6%	26.2%	
	Secondary	40.1%	37.3%	41.8%	39.9%	45.8%	34.8%	47.1%	
	High	42.8%	47.6%	37.6%	51.4%	33.9%	42.6%	26.7%	
Work-related characteristics	Workplace atmosphere								**.000**
	Negative	11.2%	6.7%	15.2%	7.2%	20.3%	14.4%	10.1%	
	Neutral	31.0%	18.6%	37.5%	25.5%	38.1%	42.8%	52.3%	
	Positive	57.8%	74.7%	47.3%	67.3%	41.6%	42.9%	37.7%	
	Size of the company								.081
	Small	27.8%	28.6%	25.4%	33.1%	17.5%	31.1%	27.8%	
	Medium/large	32.8%	33.5%	34.3%	28.6%	43.6%	26.7%	29.2%	
	Type of industry								.240
	Private	44.5%	40.8%	49.2%	45.5%	46.9%	39.7%	46.8%	
	Public	34.9%	39.0%	30.1%	33.6%	34.0%	38.7%	31.2%	
	Income (euros)								.280
	Mean	4855,14	5146,82	4666,20	4984,98	4625,45	4547, 33	4630,58	

^*^p-value of Wald statistic; p < .05

^**^Based on the highest level of education completed. ‘Low’ (primary school, intermediate secondary, US: junior high school), ‘secondary’ (higher secondary education/preparatory university education, US; senior high school; intermediate vocational education, US; junior college), and ‘high’ (higher education, US: college; university)

The *positive* class (LC 1) contained more workers who have positive experiences with workers with mental illness (45.3%), and more workers who have a work related association with mental illness (80.7%). Workers in the *moderately positive* class (LC 3) were more likely to be female (67.6%).

The positive classes (LC 1 and LC 3) contained more workers with a higher educational level (47.6%; 51.4%) and scored the highest on positive workplace atmosphere (74.7%; 67.3%).

Workers in the negative classes (LC 2 and LC 4) were more likely to have negative experiences with workers with mental illness (10.1%; 15.0%). Both classes (LC 2 and LC 4) contained less workers who have a work related association with mental illness (65.1%; 61.3%), were more likely to be male (52.2%; 56.6%) and scored lower than the other classes on higher educational level (37.6%; 33.9%). The *negative* class (LC 4) contained more workers who reported a negative workplace atmosphere (20.4%) or neutral workplace atmosphere (37.1%). Workers in the *half negative class* (LC 2) reported were more likely to report a neutral workplace atmosphere (37.5%).

The *unknowing* class (LC6) differentiated from other classes by containing the most worker with no personal experience with workers with mental illness (48.2%), by having less workers who have a work related association with mental illness (63,8%). The workers of the *partly unknowing* class (LC 5) were more likely to be female (69.5%). The workers in the *unknowing* class (LC 6) scored lower on higher educational level (26.7%). The unknowing classes (LC 5 and LC 6) contained the most workers who reported a neutral workplace atmosphere (42.8%; 52.3%).

## Discussion

The aims of this study were to examine (1) workers’ expectations of workplace mental illness disclosure outcomes, and to evaluate their expectations regarding which factors are of influence on disclosure outcomes, (2) to identify distinct subgroups of workers based on their expectations of workplace mental illness disclosure outcomes, and (3) to characterize these subgroups in terms of personal, sociodemographic, and work-related characteristics. First, the large majority of Dutch workers expected predominantly positive outcomes of workplace mental illness disclosure, despite the fact that a large majority of workers simultaneously expected disclosure to lead to advancement-related discrimination. When investigating different circumstances, workers would generally advise a friend to disclose mental illness to a manager. Second, six distinct subgroups of workers were identified based on their expectations of workplace mental illness disclosure outcomes: two positive classes (50.1% of the workers), two negative classes (33.3% of the workers), and two unknowing classes (16.7% of the workers). Third, significant differences between the six classes were found on personal experience, work-related association with mental illness, gender, educational level, and workplace atmosphere. No significant differences were found between the classes in actual having a mental illness compared to those who had not.

The findings show that respondents expected both positive and negative consequences of disclosure, which underlines the complexity of the disclosure decision. Nevertheless, overall respondents tended to be optimistic about the outcomes of disclosure, for instance illustrated by the finding that in 11 out of 14 different circumstances, they would advise a good friend to disclose. The tendency for optimism found in this study was also found in two Dutch studies, where about 75% of workers with [19] and without [20] mental illness indicated they actually had or would disclose, respectively. The contradictory finding that respondents were generally positive in their expected disclosure outcome yet believed it could lead to discrimination in the form of job loss or career advancement is puzzling and needs to be studied in future research. It could be that respondents value aspects related to worker well-being (e.g., being able to be one’s authentic self at work, or improved relationships with colleagues) higher to aspects like getting a new contract or a promotion. An additional finding that warrants further research is that about one in four respondents expected that workers who disclose would be liked less by others in the workplace and would be invited less often to social activities outside the workplace. More research into why disclosers would be liked less is needed. Apparently, workers with mental illness have a negative reputation. This was also found in a representative study of 670 Dutch line managers, where 40% indicated they were concerned that hiring a worker with mental illness would negatively affect the workplace atmosphere [7]. Finally, future research should also make clear if the findings are generalizable to other countries, or rather specific to the Dutch context and culture.

The workers in the positive classes were higher educated and scored the highest on positive workplace atmosphere, in the *positive* class workers had more positive experiences with workers with mental illness and the *moderately positive* class contained more women. In previous research, a higher educational level was found to be associated with less negative disclosure outcomes [15]. Workers with a higher educational level tend to have more knowledge about and experience with mental illness, and therefore are less likely to endorse stigmatizing attitudes [39]. Workers who perceive a positive workplace atmosphere are more likely to feel psychological safe [40], this might explains why in the current study these workers were more likely to be found in the positive classes, than in the negative classes.

Workers in the negative classes had more negative experiences with workers with mental illness, were lower educated, more likely to be male, less likely to have a work related association with mental illness and experienced a negative or neutral workplace atmosphere. Following the contact theory interacting with other groups in most cases will lead to more positive attitudes towards that group [41]. More specifically, people are more likely to diminish stigmatizing attitudes and discriminating behaviors when they interact with people with mental illness under certain conditions [42]. But not all contact lead to more positive attitudes, negative contact typically relates to situations where people were threatened and did not freely choose to have the contact [43]. This may be the case for the workers in the negative classes, after all workers did not choose their own colleagues. The differences in gender between the positive and negative classes may be explained by research indicating that men were more likely to internalize stigma than women [44, 45]. Another study showed that men, compared to women, were less likely to accept others with mental illness [46].

Workers in the unknowing classes were more likely to report a neutral workplace atmosphere, and the *unknowing* class contained more workers with no experience with workers with mental illness. It is not surprising that people who had no experience working with workers with mental illness indicated that they did not know what to expect of workplace mental illness disclosure outcomes.

Within five classes a majority expected that disclosure would lead to an increased chance of no renewal of a temporary contract, still the majority indicated that they would disclose or disclosed a mental illness. However, discrimination seems to be a realistic threat, findings from another Dutch study showed that a majority of line managers was reluctant to hire workers with mental illness [7]. One of the reasons why Dutch workers tend to be open could be that Dutch workers are well protected by legalization [34], which may give workers the idea that it is safe to disclose a mental illness. Whether disclosure is preferable for a worker is a complex and individual choice, and the outcome is influenced by many factors [1].

### Strengths and limitations

The strengths of this study are the use of a large sample of Dutch workers from the LISS-panel, representative of those with paid work and who were not working in management positions. For this panel workers were recruited based on a true probability sample from population registers and workers were allowed to participate anonymously, which reduces the risk of social desirability bias [47]. In addition, this dataset is one of the first datasets that focusses on workplace stigma in the Netherlands, it gives reliable new insights in the considerations of Dutch workers regarding disclosure. Limitations of this study are that the data are self-reported, and that they were based on hypothetical circumstances and perceptions, rather than actual behavior. Future studies should also focus on actual outcomes of workplace disclosure. Nevertheless, perceptions are important predictors of actual behavior [48], and many workers fear that disclosure will lead to discrimination (e.g., job loss, no contract renewal, lower wages) [1, 30, 49]. Whether or not these perceptions are true, they can influence workers’ disclosure behavior (non-disclosure). For example, two previous studies of Dutch workers with and without mental illness showed that 27% had not disclosed [19] and 26% would not disclose [20], respectively, and that fear of negative career consequences was an important reason for non-disclosure. Importantly however, non-disclosure also means that these workers miss out on potential workplace support or workplace accommodations, which can be of crucial importance to prevent adverse outcomes such as sick leave, a worsening of health problems or job loss. An additional limitation is the cross-sectional design of this study, for which causality cannot be claimed. Future research on workplace mental illness disclosure decisions with a longitudinal design are needed. Another limitation is that part of the questions referred to employee's perceptions in general, whereas others evaluated what the employee would advise a close friend under certain circumstances.

## Conclusion

In conclusion, this study shows that most of the Dutch workers were predominantly positive about outcomes of workplace mental illness disclosure, even though a high number of respondents also expected substantial negative outcomes, such as that workplace disclosure might lead to advancement-related discrimination by employers. This study identified six different classes based on their expected workplace mental illness disclosure outcomes: two positive, two negative and two unknowing classes. Our results indicated that workers differ in their expected workplace disclosure outcomes based on differences in personal experience, work-related association with mental illness, gender, educational level, and workplace atmosphere.

The insight into the six classes and their characteristics obtained in this study illustrates there are important differences in workers’ expectations. With positive *and* negative outcome expectations both being highly prevalent, the findings stress the complexity of the disclosure decision and the importance of more research in this area, preferably including not only expectations but also actual disclosure outcomes. Furthermore, insights into workers’ expectations contribute to a new and understudied research field that ultimately can have important implications for practice, as the disclosure decision process plays an important and underestimated role in the sustainable employment and well-being [16, 28, 29, 50]. As this is a novel area, high quality studies on how to adequately support workers with (mental) health problems in their decision-making process are needed, like studies on how peer support can help in the decision-making process [6]. This is important, as 27% of Dutch workers does not disclose mental illness in the work environment, and therefore by definition also misses out on workplace support and accommodations that may be important to stay at work. Finally, the findings add to the growing literature that a safe workplace atmosphere, where workers with health problems are supported rather than excluded, is not only highly important for disclosure, but ultimately also for workers’ sustainable employment, health and well-being [1, 19, 49, 51]

## Data Availability

Data are available on reasonable request. The data set used is available from the corresponding author on reasonable request.
